# Malignant Perivascular Epithelioid Cardiac Sarcomas: A Case Report and a Review of the Literature

**DOI:** 10.1155/2015/258140

**Published:** 2015-04-22

**Authors:** Candice Baldeo, Abdul wahab Hritani, Robert Ali, Sana Chaudhry, Fawad N. Khawaja

**Affiliations:** ^1^Department of Internal Medicine, University of Florida, Jacksonville, FL 32209, USA; ^2^Department of Cardiothoracic Surgery, University of Florida, Jacksonville, FL 32209, USA

## Abstract

Cardiac tumors, either benign or malignant, are difficult to diagnose due to their rarity, variety, and nonspecific presentation. Since primary cardiac sarcoma remains an unusual diagnosis, the literature on its presentation, diagnosis, and optimal management remains scarce. To our knowledge the following case of cardiac perivascular epithelioid cell tumor is the fourth reported case found in the literature. Although complete surgical resection remains the gold standard for cardiac sarcomas, our case demonstrates that not all of them can be completely resected.

## 1. Introduction

The prevalence of primary cardiac tumors is 0.001–0.03% in autopsy series, with malignant tumors accounting for 25% of cases [1]. Seventy-five percent of primary tumors are benign in origin, with myxoma being the most frequent in over 50% of cases. From the 25% of malignant cardiac tumors, the most frequent are cardiac sarcomas [1].

Resection remains the primary mode of treatment for primary cardiac sarcomas (PCSs). However, PCS usually has a high recurrence rate of up to 50% even after resection, and the prognosis remains dismal [2]. Early diagnosis and initiation of treatment, resection and/or chemotherapy with radiation therapy, may decrease recurrence and have mortality benefit.

## 2. Case Description

A 64-year-old Caucasian female with a past medical history significant for hypertension and hyperlipidemia presented to our institution with progressive shortness of breath on exertion, bilateral lower extremity edema, and a chronic dry cough for the past 3 months. She also complained of a decreased appetite and a 12-pound weight loss over the past 2 months. She denied orthopnea or paroxysmal nocturnal dyspnea. She had no previous smoking history and no significant surgical or family history. Physical examination revealed sinus tachycardia, bilateral pitting pedal edema, and basilar crackles in both lungs. Laboratory diagnosis showed leukocytosis (13.2), microcytic anemia (10.5 Hb, MCV 78.5), and thrombocytosis (524).

**Figure 1:**
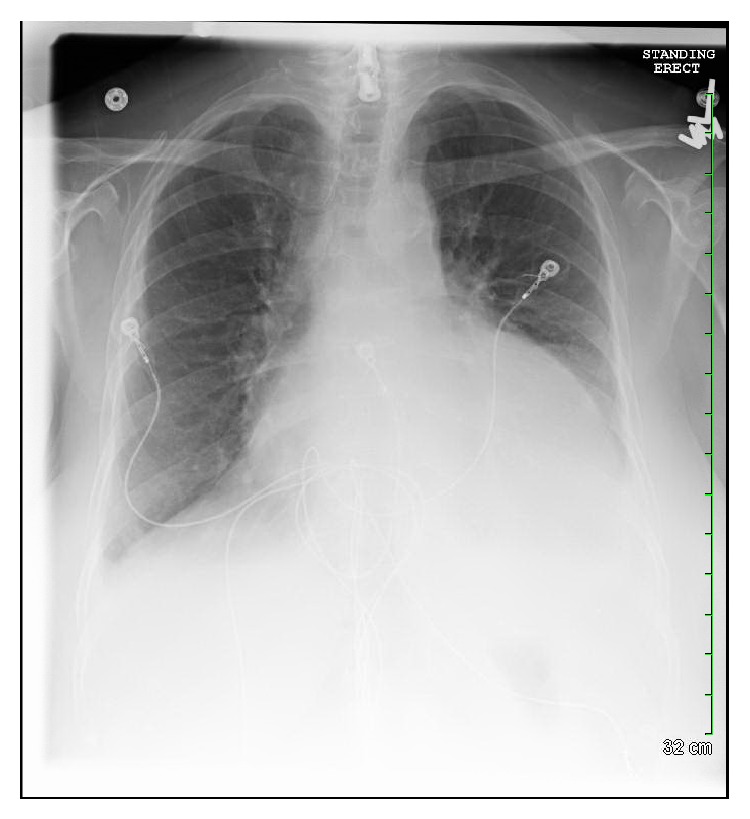
PA CXR showing cardiomegaly, interstitial edema, and small bilateral effusions.

**Figure 2:**
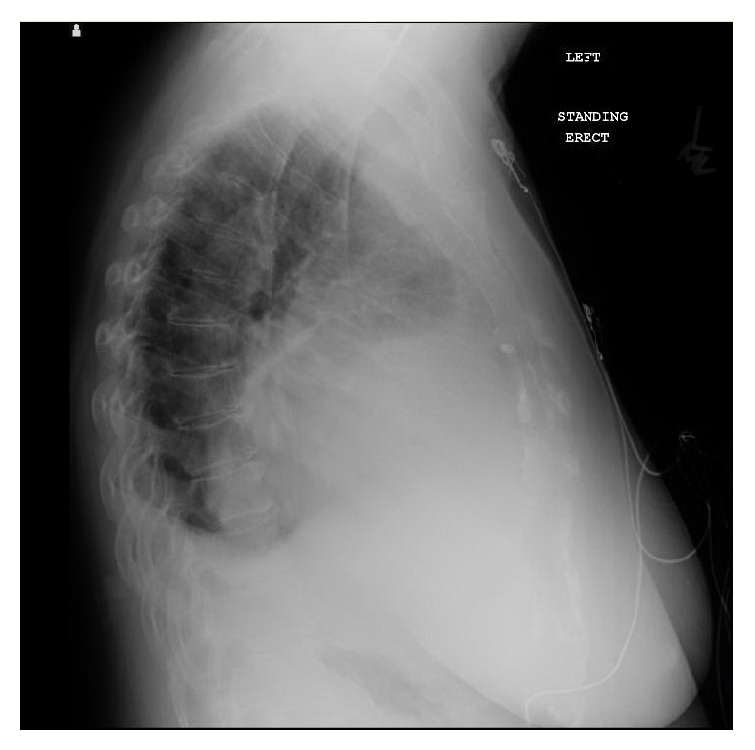
Lateral CXR showing cardiomegaly with loss of retrosternal airspace.

**Figure 3:**
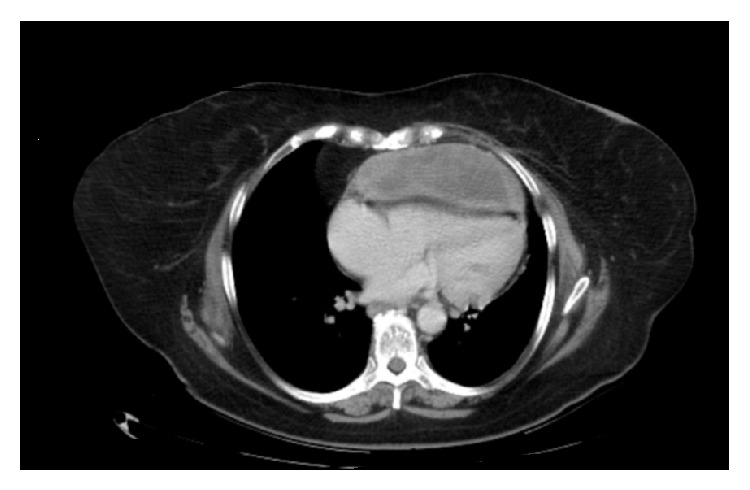
CT of chest showing 9.9 × 11.5 × 14.2 cm heterogeneous mass, with internal necrosis and calcification, associated with the anterior pericardium and displacing the heart superiorly and posteriorly.

Chest X-ray displayed cardiomegaly with increased interstitial pulmonary markings and small bilateral pleural effusions (see Figure 1). On lateral chest X-ray there was loss of the retrosternal airspace (see Figure 2). Low voltage QRS with sinus tachycardia was noted on the EKG. Computed tomography (CT) of the chest without contrast revealed a 9.9 cm × 11.5 cm × 14.2 cm heterogeneous mass located along the anterior pericardium which was significantly displacing the heart superiorly and posteriorly (see Figure 3). This mass also exhibited internal necrosis and calcifications. Transthoracic echocardiogram (TTE) showed a preserved ejection fraction with a large mass which was compressing the anterior right ventricle.

**Figure 4:**
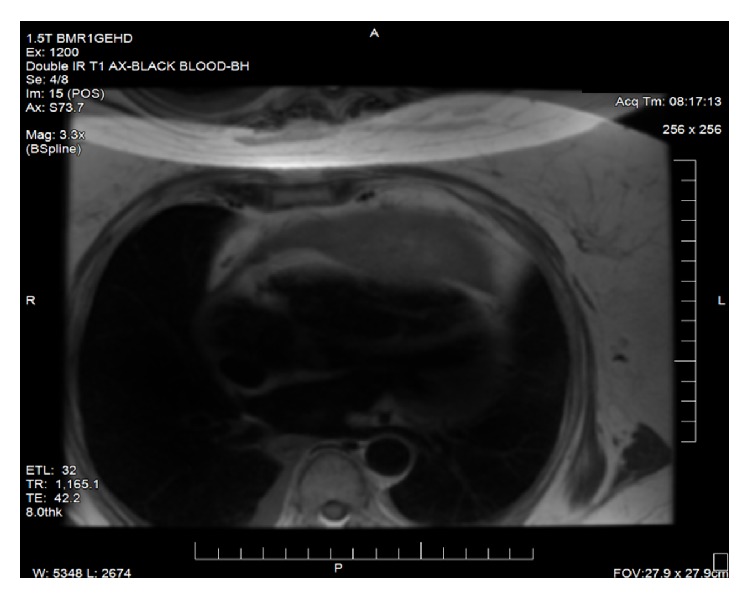
Cardiac MRI showing cystic solid mass arising in the pericardium.

Cardiac magnetic resonance imaging (CMRI) showed that the mass was cystic and solid in nature and again arising near anterior pericardium (see Figure 4). A CT guided core needle biopsy was performed and sent to pathology where it revealed spindle and epithelioid sarcoma. Immunostains on tumor biopsy were positive for SMA and calretinin (focal in epithelioid nests) and negative for cytokeratins (Ck-AE1/AE3 and CK5/6), TTF-1, mammaglobin, breast gross cystic disease fluid protein-15, estrogen/progesterone receptors, CD34, and desmin. Left heart catheterization showed no significant abnormalities.

After discussion with the patient and her family, the decision was made to perform surgery for a complete resection of the tumor. However, at surgery it was discovered that the mass was actually arising from the pericardium and wedged underneath it. Attempts to debulk the tumor demonstrated that it had already infiltrated the myocardium. Thus, the cardiac tumor was deemed unresectable.

**Figure 5:**
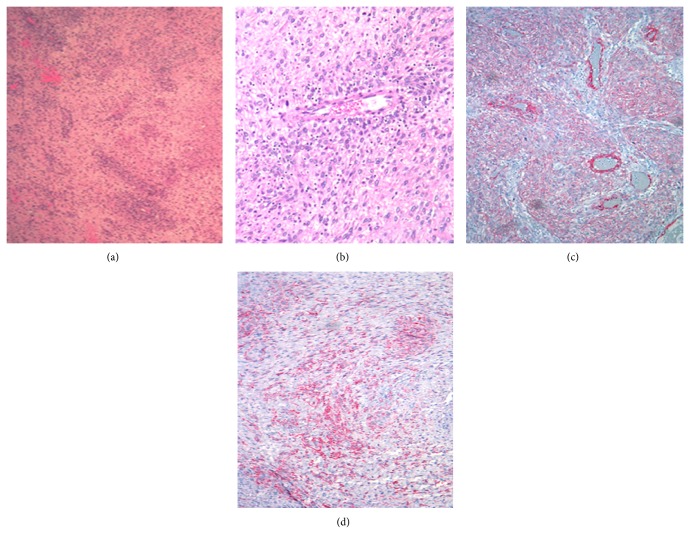
Perivascular condensation of tumor cells (H&E stain (a) ×2.5 and (b) ×20). Diffuse positivity for SMA ((c) ×10) and HMB-45 ((d) ×10).

Pathology results of the partial tumor excision showed high-grade sarcoma with features favoring malignant perivascular epithelioid cell tumor (see Figure 5). The debulked tumor fragments (measuring collectively 12.2 × 10.8 × 4.0 cm) were grey to yellow white solid with pink-brown ragged cystic areas. Histologically, the tumor was formed of poorly differentiated high-grade spindle and epithelioid cell sarcoma with moderate nuclear pleomorphic and scattered multinucleated tumor giant cells. Mitoses were brisk with abnormal figures (>30 mitoses/10 HPF). The tumor architecture ranged from compact fascicles to cords and strands in loose edematous to myxoid and sclerotic stroma. There was prominent perivascular tumor cells condensation around thin wall blood vessels. Large areas of geographic necrosis were present in about 45% of sampled tumor. Additional stains on the debulked tumor tissue revealed focal strong positivity for desmin, SMA, HMB-45, melan A, and S100 and strong diffuse positivity for CD99 (membranous) and BCl-2. The immunostain results were in favor of PEComa.

Postoperatively, the patient required vasopressor support and intubation for respiratory failure. Vasopressors were weaned over the following 48 hours and she was eventually extubated. She refused any further chemotherapy or radiotherapy and opted for home with hospice care. She died 6 months following surgery.

## 3. Discussion

Primary cardiac sarcomas may occur in any chamber of the heart. Clinical presentation is often nonspecific and depends upon the location of the tumor. Patients can present with generalized symptoms including subjective fevers, weight loss, generalized weakness, and fatigue. Additionally, tumors can infiltrate the myocardial wall and lead to cardiomyopathy and heart failure symptoms as in our case. Furthermore, tumor infiltration into the neural pathways can cause arrhythmias and atrioventricular block. Also, intracavitary tumors, that is, when they protrude inside the atrium or the ventricle, can result in cerebrovascular accidents and pulmonary emboli when they become detached. In some rare cases, the first manifestation of a cardiac tumor is sudden cardiac death [2].

Generally, clinical history and echocardiography can help the diagnosis in most cases. Lab findings can be nonspecific and include leukocytosis, anemia, thrombocytosis, and elevated ESR. Echocardiography represents a substantial imaging technique for the detection of cardiac tumors with high sensitivity and specificity (90% and 95%, resp.) [1]. Newer techniques that may aid in the differential diagnosis of cardiac tumors from other cardiac masses include contrast echocardiography, 3D echocardiography, cardiac MRI, and cardiac CT. Definitive diagnosis is established by histopathology [1].

Surgery remains the backbone of management for cardiac sarcomas [3]. The most important factor dictating prognosis is the ability to achieve a complete surgical resection of the tumor. It has been observed that the median survival increases to 27 months with complete resection compared to 10 months where complete resection is not possible [4]. Surgery not only alleviates symptoms but also confirms diagnosis and avoids future embolic and hemodynamic complications [5]. However, complete macroscopic resection is possible in only 33% of patients [5]. Even in patients with apparent complete excision, high recurrence rates have been reported. After surgical intervention, adjuvant radiotherapy for local recurrence and chemotherapy for control of systemic disease are advised [5].

The response of soft tissue sarcomas to radiation has been well documented and currently adjuvant radiation is recommended along with surgical resection to improve overall survival [3]. Chemotherapy is currently reserved for metastatic tumors and the agents of choice are doxorubicin and ifosfamide, with response rates ranging from 55 to 66% [3]. In cardiac sarcomas, the use of chemotherapeutics may extend to the treatment of the primary tumor if surgery is ineffective [3].

Unfortunately, survival from the time of diagnosis varies from 7 months to a maximum of 2 years and there is insufficient evidence to define optimal treatment [2, 6, 7]. On long-term follow-up, the majority of patients die of distant metastases [2, 6, 7]. The average survival of patients treated purely conservatively with chemotherapy and radiotherapy is just under a year [2]. Simple, incomplete resection (debulking) extends survival by only a few months [2, 6, 7].

Heart transplantation can occasionally be considered as a final treatment option in individual cases, provided distant metastases can be ruled out [2, 8]. One significant risk of this procedure is the danger of exacerbation of undetected micrometastases by the necessary immunosuppression [2].

## 4. Perivascular Epithelioid Cell Tumor (PEComa)

Perivascular epithelioid cell tumor (PEComa) is recognized as a very rare mesenchymal tumor composed of histologically and immunohistochemically distinctive perivascular epithelioid cells [9, 10]. Perivascular epithelioid cell tumors have now been reported in almost every body site, and the growing list of reported sites includes gastrointestinal site, gynecologic site, genitourinary site, extremities, and skin, as well as single reports in the heart, breast, oral cavity, orbit, and skull base [9]. PEComas with a size >8 cm and mitotic counts of 1/50 HPF are categorized as malignant, such as our case [10]. Precise rates for survival, metastasis, and recurrence specific to primary malignant perivascular epithelioid cell tumor are still not available.

A number of the morphologic features of PEComas, including the admixture of spindled and epithelioid forms, the occasionally prominent nucleoli, and the presence of multinucleated cells, are also seen in melanoma and clear cell sarcoma. PEComas can histopathologically be confused with carcinomas, smooth muscle tumours, adipocytic tumours, and gastrointestinal stromal tumours (GIST) [11].

Based on our literature research, this perivascular epithelioid cell tumor is the fourth reported case found in the heart [9]. The previous cases were 2 young males (aged 29 years and 20 years, resp.) and a 10-year-old girl, and the tumors were overtly malignant only in the adult cases.

Geramizadeh and colleagues [10] described a case of a 20-year-old man who presented with chest pain, palpitation, and dyspnea for 4 days. A TTE showed a cardiac mass and decreased ejection fraction. Spiral chest computed tomography (CT) scan with contrast showed a giant mass located in the posterior part of the pericardial cavity surrounded by pericardial effusion. At surgery, the mass was detected at the posterior aspect of the heart with adhesion to the right and left ventricle and right atrium and was completely resected. On gross examination, the tumor was 15 × 14 × 6 cm. Pathology showed that the mass was a malignant perivascular epithelioid cell tumor (PEComa). The patient refused to receive chemotherapy or radiation and survived 6 months after surgery.

Tazelaar et al. described a case of a 29-year-old man with a seven-year history of atrial fibrillation presented with symptoms of mitral stenosis [12]. Thoracotomy revealed a left atrial tumor growing through the interatrial septum to involve right atrium and extending throughout the left lateral wall into the pericardial sac. Grossly, the tumor appeared to extend very near the pulmonary veins. Because of its infiltrative nature, only incisional biopsies were taken at initial operation. Subsequently, the tumor was completely resected and left atrial reconstruction performed with a bovine graft and reimplantation of pulmonary veins. The patient died shortly thereafter of heart failure thought to be due to nontumorous coronary artery thromboemboli. At postmortem examination, no residual primary or metastatic tumor was found.

Tai et al. reported a case of cardiac PEComa in a 10-year-old girl who had presented with 8 years of cardiac murmur and 2 weeks of increasing dyspnea. Chest computed tomography showed a solid mass located in the left atrioventricular groove. At surgery a 5.5 × 3 × 4.8-cm bulging mass was detected in the left atrioventricular groove. The base of the neoplasm was fixed in the posterolateral wall of the left cardiac ventricle. The slightly firm tumor was well defined with a smooth external surface. The tumor protruded from the cardiac wall into the pericardial cavity with massive effusion. A total tumorectomy was performed, and the postoperative course was uneventful. The patient is alive and well without evidence of disease 18 months after surgery [9].

## 5. Conclusion

When a cardiac tumor is confirmed, it is crucial for oncologists and surgeons to collaborate. These tumors are of a particular concern due to the fact that overt signs and symptoms occur rather late in the course, precluding effective tumor eradication as in our case. Perhaps if neoadjuvant therapy was attempted, complete surgical resection may have been possible.
